# Gamma-enolase: a well-known tumour marker, with a less-known role in cancer

**DOI:** 10.1515/raon-2015-0035

**Published:** 2015-08-21

**Authors:** Tjasa Vizin, Janko Kos

**Affiliations:** Faculty of Pharmacy, University of Ljubljana, Ljubljana, Slovenia

**Keywords:** gamma-enolase, cancer, glycolysis, cell survival, tumour marker

## Abstract

**Background:**

Gamma-enolase, known also as neuron-specific enolase (NSE), is an enzyme of the glycolytic pathway, which is expressed predominantly in neurons and cells of the neuroendocrine system. As a tumour marker it is used in diagnosis and prognosis of cancer; however, the mechanisms enrolling it in malignant progression remain elusive. As a cytoplasmic enzyme gamma-enolase is involved in increased aerobic glycolysis, the main source of energy in cancer cells, supporting cell proliferation. However, different cellular localisation at pathophysiological conditions, proposes other cellular engagements.

**Conclusions:**

The C-terminal part of the molecule, which is not related to glycolytic pathway, was shown to promote survival of neuronal cells by regulating neuronal growth factor receptor dependent signalling pathways, resulting also in extensive actin cytoskeleton remodelling. This additional function could be important also in cancer cells either to protect cells from stressful conditions and therapeutic agents or to promote tumour cell migration and invasion. Gamma-enolase might therefore have a multifunctional role in cancer progression: it supports increased tumour cell metabolic demands, protects tumour cells from stressful conditions and promotes their invasion and migration.

## Introduction

Enolases (EC 4.2.1.11) are intracellular enzymes that catalyse the dehydration of 2-phospho-D-glycerate to phosphoenolpyruvate in the catabolic direction of the glycolytic pathway, a process converting glucose into pyruvate, which enables the formation of high-energy compounds of ATP and NADH. In the anabolic direction during gluconeo-genesis, they catalyse the reverse reaction of hydration of phosphoenolpyruvate to 2-phospho-D-glycerate. The glycolytic pathway and its enzymes are one of the most conserved and important metabolic networks in living organisms and therefore, enolases are among the most ubiquitously and abundantly expressed proteins.^1^–^4^ Despite being expressed in most cells, the gene that encodes for enolase is not a housekeeping gene since its expression varies during several developmental, metabolic or pathophysiological conditions.^5^ In addition to their innate glycolytic function, many enzymes of the glycolytic pathway, including enolase, were shown to possess various specific regulatory functions and to play a pleiotropic role in physiological and pathological processes, including cancer.^1^,^2^,^6^,^7^ In this paper we review the properties, distribution and function of gamma-enolase and its role in enhanced glycolysis and proliferation of tumour cells. Additionally, we expose new mechanisms through which gamma-enolase may promote cancer progression: aiding adaptation of tumour cells to stressful conditions by activating survival promoting signalling pathways and promoting migration of tumour cells. Finally, we discuss the role of gamma-enolase as a marker of exposure to carcinogenic pollutants and review the diagnostic and prognostic utility of gamma-enolase in cancer patients.

## Properties and distribution of enolase

Enolases are functionally active as dimers, composed of non-covalently linked subunits alpha- (α), beta- (β) and gamma- (γ), facing each other in an antiparallel fashion, which may form five homodimeric or heterodimeric isoenzymes, expressed in a development and tissue-specific manner. The iso-enzyme αα (alpha-enolase) is localized in all foetal and in the majority of adult mammal tissues. During tissue development, it is replaced by other isoforms: in skeletal and heart muscles by αβ and ββ (beta-enolase), and in neuronal cells and cells of the diffuse neuroendocrine system by isoenzymes αγ and γγ (gamma-enolase). In mammals, each of the three isoenzymes is encoded by an independent loci.^8^,^9^ All enolase isoforms have a molecular range between 82 and 100 kDa and share high sequence identity and kinetic properties.^1^,^6^,^10^–^12^ However, each isoform possesses characteristic short variable regions, which are situated predominantly on the surface of the molecule and might be the sites of contact with different cytoskeleton elements or other cell components.^13^

Besides the peptide molecule, enolase requires a divalent metal ion for its stabilisation and catalytic activity. Six divalent metals have been demonstrated to activate enolase: Mg^2+^, Zn^2+^, Cd^2+^, Co^2+^, Mn^2+^ and Ni^2+^. The most abundant is Mg^2+^, which provides the highest activation strength.^1^,^14^,^15^ The metal ion is not firmly bound into the protein part of the molecule; therefore enolase is not a typical metalloenzyme, but defined as a “metal-ion-activated enzyme complex”.^16^ Enolase has two binding sites for Mg^2+^, both contributing to catalysis: binding to the first site, Mg^2+^ induces conformational changes of the active site enabling the binding of the substrates, whereas the binding of a second Mg^2+^ is an essential part of the catalytic apparatus.^1^,^17^–^19^

Enolase localizes predominantly in the cytosol however, variations in cellular localisation were observed for all three enolase isoforms. Alpha-enolase was observed in the nucleus, on the cell surface and in extracellular space. It may interact with different cytoplasmic, nuclear and membrane molecules and exhibits several other functions besides catalysis.^1^,^20^ The nuclear form of alpha-enolase was recognized as Myc promoter-binding protein-1 (MBP-1), an alternative splicing form involved in regulation of transcription by repressing the function of Myc and acting as a tumour suppressor.^6^,^21^–^23^ Alpha-enolase localizes also on cell surface of neuronal, endothelial and hematopoietic cells as well on pancreatic, breast and lung cancer cells. Its surface expression was shown to depend on the pathophysiological conditions of the cells and its C-terminal lysine residue acts as a plasminogen-binding receptor modulating peri-cellular fibrinolytic activity and promoting migration and metastasis of cancer cells. The cell surface alpha-enolase is catalytically active, maintaining its active dimeric form. Alpha-enolase was shown also to be secreted from cells by exosomes, cell derived vesicles, proposed to play an important role in intercellular communication.^23^–^25^ However, the mechanisms of surface translocation, membrane attachment, cell surface expression or secretion remain unknown.^6^,^26^–^32^ The properties and function of alpha-enolase in malignant disease have been extensively studied and reviewed.^1^,^2^,^6^,^20^,^23^

Different subcellular localisation and interactions with other proteins were observed also for beta-enolase during maturation, normal function and regeneration of muscles. Specific interactions with macromolecules may address beta-enolase to the subcellular site where ATP, produced through glycolysis, is most needed for muscular contraction or regeneration.^33^–^35^ Increased expression of beta-enolase was detected in rhabdomyosarcoma tissue, which is, to our knowledge, the only evidence that this isoform might be involved in cancer.^36^,^37^

## Gamma-enolase

Gamma-enolase, is a 433 amino acid long acidic dimeric protein, which includes two enolase isoenzymes, γγ and αγ, and is also referred as neuron-specific enolase (NSE). The subunit molecular mass is approximately 39 kDa, whereas Mr of the native form is 78 kDa which might vary on the subunit combination. Gamma-enolase localizes predominantly in neuronal cells and in neuroendocrine cells, particularly in those of the amine precursor uptake and decarboxylation (APUD) lineage, for example in the intestine, lung, thyroid and pituary gland and pancreas.^8^,^38^ It is found in lower amounts also in non-neuronal and non-neuroendocrine tissues or cells, such as erythrocytes, platelets, breast tissue, prostate and uterus.^39^–^41^ The γγ isoform is found predominantly in mature neurons and is also used as marker of neuronal maturation and differentiation, while the αγ isoenzyme localizes in higher amounts in non-neuronal cells.^8^,^9^

**FIGURE 1.**
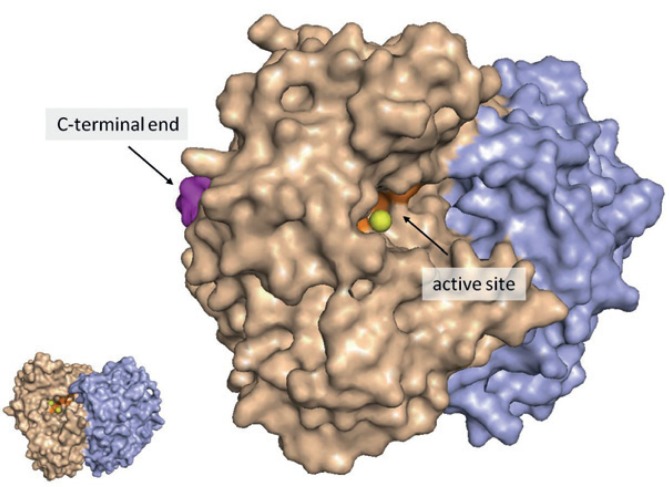
Position of gamma-enolase catalytical active site and the PDZ-binding motif containing C-terminal end. Subunits of the γγ-dimer are represented by separate colours (wheat and violet). The orange part represents the catalytical active site, yellow balls represent Mg^2+^ ions and the magenta part represents the C-terminal end of the molecule (the last 6 amino acids). For better representation, active site and C-terminal end are shown only in one subunit. The image was created using PyMOL (DeLano LLC Scientific). Gamma-enolase crystal structure (1TE6) was obtained from Protein Data Bank (PDB). The image was prepared by authors and has not been published elsewhere.

The C-terminal end of gamma-enolase contains a PDZ-binding motif (431S-433L: SVL) (Figure 1), which might enable an interaction with several proteins that contain a PDZ-domain and are involved in intracellular redistribution of molecules and signalling pathway events. Different gamma-enolase cellular localisation, which depends on the pathophysiological conditions of the cells, propose other cellular engagement besides glycolysis. In neuronal, glial and astrocytic cells, gamma-enolase was shown to associate with the plasma membrane, or even appear on the surface of cells^42^–^45^, which might occur through its hydrophobic domain in the N-terminal region (32A-43Y: AAVPSGASTGIY). Also, on the cell surface alpha-enolase may bind to plasminogen by C-terminal lysine.^46^ In contrast to alpha-enolase, gamma-enolase has no C-terminal lysine and does not bind plasminogen; therefore it might exert other functions on cell surface.^43^,^46^–^48^ Gamma-enolase was detected also in the nucleus of malignantly transformed urothelial and epithelial breast cells and in glioblastoma cells; however its role remains unknown.^40^,^49^–^51^ Significantly higher increase of gamma-enolase antigen levels than its catalytical activity was observed during exponential growth of small-cell lung cancer cells, proposing that cellular gamma-enolase exists also as an enzymatically inactive compound, that might possess other functions.^52^

## The function of gamma-enolase in increased glycolysis in cancer

It is generally known that glycolysis is drastically enhanced in tumour cells and is a hallmark of cancer progression.^53^,^54^ In tumours that outgrow its feeding circulation, cells are exposed to an environment with poor oxygen and nutrients supply^50^, which leads to a prevalence of aerobic glycolysis over mitochondrial oxidative phosphorylation.^55^–^57^ This metabolic switch referred also to as the Warburg effect, enables tumour cells to produce energy to survive and eventually proliferate regardless the presence of oxygen. Glycolysis alone, however, is energetically less efficient than oxidative phosphorylation. Therefore, reactions of the glycolytic pathway have to be drastically accelerated to satisfy the higher metabolic needs of proliferating tumour cells, which is evident from a net increase in glucose consumption and higher expression of glycolytic enzymes.^55^,^58^–^60^

Gamma-enolase is overly-expressed in tumours^39^ and its major contribution to tumour progression is, no doubt, the participation to accelerated glycolysis of cancer cells. For instance, malignant transformation of astrocytic^61^, breast^40^ and urothelial cells^49^ led to occurrence of gamma-enolase in originally gamma-enolase-negative cells and to colony formation and proliferation, which strongly suggests that transformed cells might obtain the ability to express gamma-enolase in order to adapt to increased metabolic needs of a neoplastic state.^61^,^62^ Further, malignantly transformed urothelial cells, which were able to proliferate and form tumours when inoculated into immune compromised mice, were shown to express higher levels of gamma-enolase, compared to less active and differentiated cells. Authors proposed that cells, which express gamma-enolase at higher rates, might have an advantage in tumour initiation and subsequent growth.^49^ Gamma-enolase was significantly up-regulated also in glioblastoma cells exposed to hypoxia and serum starvation, and additionally, its knock-down significantly diminished cell growth^50^, supporting the findings that the dependence of tumour cell growth on glycolysis is even more emphasized in stressful conditions.^55^,^60^,^63^ Finally, in non-small cell lung cancer cells, an alternative splicing form of c-H-ras, p19^ras^, was shown to specifically bind gamma-enolase and inhibit its enzymatic activity, resulting in diminished cell proliferation.^58^ The glycolytic function of gamma-enolase and its impact on promoting tumour cell growth represents a promising target for cancer therapy.^64^

## The pro-survival function of gamma-enolase in cancer

Gamma-enolase was shown to act as a neurotropic factor in neuronal cells.^7^,^65^,^66^ This function is manifested through an additional active site, which is not a part of the catalytical apparatus involved in glycolysis, but localized at the C-terminal end of the molecule. For instance, a 30 amino acid long peptide, mimicking the C-terminal part of gamma-enolase, was shown to promote survival, differentiation and regeneration of neurons by activating signal transduction pathways which are normally triggered by the activation of Trk receptor: phosphatidylinositol 3-kinase (PI3K) and mitogen-activated protein kinase (MAPK) pathways. Additionally, the C-terminal peptide of gamma-enolase was demonstrated to impair apoptosis and to interact with p75 neurotrophin receptor (p75^NTR^) and suppress the activation of its downstream effectors in apoptotic signalling. Despite having similar amino acid sequence in the C-terminal part, other enolase isoforms do not show a neurotropic function.^7^,^43^,^46^,^67^–^69^ Gamma-enolase neurotrophic effect is regulated by cathepsin X, a cysteine carboxymonopeptidase, which is frequently expressed in neuronal and glial cells.^70^,^71^ Cathepsin X was shown to sequentially cleave the final two amino acids (433L and 432V) at the C-terminal end of gamma-enolase and to disrupt the PDZ motif, through which gamma-enolase binds to the scaffold protein gamma-1-syntrophin. The latter mediates the translocation of gamma-enolase and its association with plasma membrane, which is a prerequisite for neurotrophic activity.^43^,^46^ Therefore, only C-terminally uncleaved gamma-enolase has a pro-survival activity. The protective function of gamma-enolase was observed also in brains of a mouse model of Alzheimer disease (Tg2576): C-terminally truncated gamma-enolase localized in immediate plaque vicinity and strongly colocalized with cathepsin X, while uncleaved gamma-enolase exhibiting neuroprotective activity, localized in microglia cells in close proximity of senile plaques. Additionally, using a mouse microglial cell model, gamma-enolase was shown to protect neuronal cells from amyloid-β peptide toxicity and cathepsin X reversed its function.^66^

**FIGURE 2.**
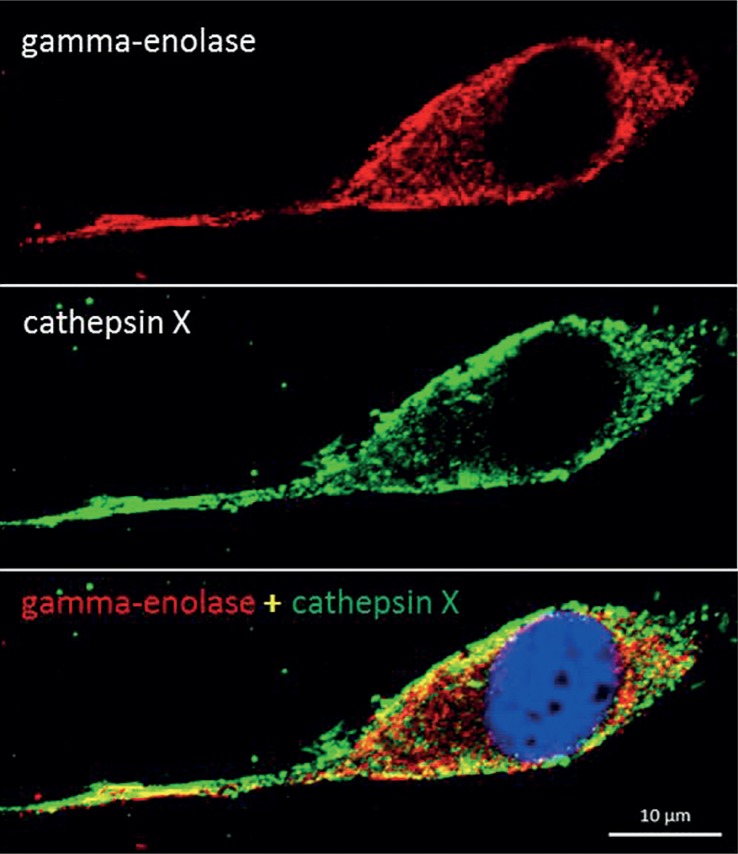
Co-localization of gamma-enolase and cathepsin X in human glioblastoma cells U87-MG grown in serum-free medium for 72 h. U87-MG cells were grown in Eagle´s Minimum Essential Medium (EMEM, Sigma), supplemented with 10% (v/v) foetal bovine serum (HyClone), 1% L-glutamine (Sigma) and 1% penicillin/streptomycin (Sigma) at 37°C and humidified atmosphere with 5% CO_2_. For protein visualization, cells were seeded on glass coverslips at a concentration of 1 × 10^4^ cells/ml in 24 well plates. After 24 h, complete growth medium was replaced with serum-free medium and cells were left to grow for additional 72 h. After treatment cells were fixed with 10% formalin for 30 min at room temperature and then permeabilized by 0.5% Tween®20 in phosphate buffered saline (PBS), pH 7.4 for 10 min. Non-specific binding was blocked with 3% bovine serum albumin (BSA) in PBS, pH 7.4 for 1.5 h at room temperature. Cells were then incubated with primary antibody against N-terminal end of gamma-enolase (10 μg/ml, goat polyclonal, Santa Cruz Biotechnology) and active cathepsin X (10 μg/ml, mouse monoclonal, 2F12) in 3% BSA in PBS pH 7.4 for 2 h at room temperature. After three washes with PBS, pH 7.4, cells were incubated with Alexa Fluor 555 donkey anti-goat (Molecular Probes™) and Alexa Fluor 488 donkey anti-mouse (Molecular Probes™) secondary antibody in 3% BSA in PBS, pH 7.4. After washing with PBS, ProLong® Gold Antifade Mountant with 4’,6-diamidino-2-phenylindole, dilactate (DAPI, Molecular Probes™) was used to mount coverslips on glass slides. Fluo rescence microscopy was performed by Carl Zeiss LSM 710 confocal microscope (Carl Zeiss Oberkochen) with ZEN 2012 image software. Gamma-enolase (red) and cathepsin X (green) staining showed co-localisation in the perimembrane region. The blue staining with DAPI represents the nucleus. The image was prepared by authors and has not been published elsewhere.

Gamma-enolase has been proposed to act as a pro-survival factor also in cancer cells. It was shown to support glioblastoma cell adaptation to cellular stress, such as serum starvation, hypoxia, chemotherapy and radiotherapy; however, no specific mechanism has yet been proposed.^50^ Both, starvation and hypoxia have been linked to progression of cancer and resistance to treatment by inducing biological changes in tumour cells, one of them being increased glycolysis.^55^,^60^,^72^ However, C-terminally uncleaved gamma-enolase might additionally support tumour cell adaptation to stressful conditions by activating survival promoting signalling pathways as it does in neuronal cells, and cathepsin X, which is present also in tumour cells^71^, might regulate its function (Figure 2). For instance, in glioblastoma cell lines, exposed to serum starvation or hypoxia, gamma-enolase expression was significantly increased^50^; moreover, significant increases in protein and phosphoprotein levels were observed also in PI3K/Akt and MAPK/ERK and anti-apoptotic signalling pathways^73^,^74^, which are triggered by gamma-enolase in neuronal cells. Separate analysis of expression and role of C-terminally uncleaved and truncated gamma-enolase in cancer cells and tumour tissue might provide new information on its involvement in tumour progression.

## The role of gamma-enolase in migration of tumour cells

Recently, a study on glioma cells showed that gamma-enolase knockdown significantly reduced migration of cells; however, no specific mechanism has been proposed.^75^ An important prerequisite for cell migration is a dynamic remodelling of actin cytoskeleton. Remodelling is stimulated by several molecules that link migratory signals to the actin filaments and are upregulated in invasive and metastatic cancer cells.^76^ In neuroblastoma cells, gamma-enolase was shown to co-localize with actin filaments, an interaction that depends on the presence of gamma-1-syntrophin.^43^ Additionally, gamma-enolase C-terminal peptide was shown to regulate RhoA kinase, a regulator of actin cytoskeleton organization. Consequently, gamma-enolase induced actin polymerisation and its redistribution to growth cones of neurites.^68^ Similarly, alpha-enolase was shown to bind to actin and tubulin^77^ and to mediate invasiveness of tumour cells^78^ and sensitivity to microtubule targeted drugs.^79^ These results provide evidence, that gamma-enolase might be involved in migration of tumour cells through interactions with actin filaments and regulation of RhoA kinase function.

## Gamma-enolase as a marker of exposure to environmental carcinogenic pollutants arsenic and cadmium

Arsenic and cadmium exposure is linked to breast and bladder cancer occurrence. Exposure of breast epithelial and urothelial cells to As^3+^ or Cd^2+^ was shown to induce malignant transformation of cells and an increase of mRNA and protein levels of gamma-enolase in the cytoplasm and nucleus of cells, while expression of alpha-enolase did not change. Authors proposed that gamma-enolase might be translated as a possible biomarker for chronic environmental exposure to As^3+^ or Cd^2+^. Its expression in non-malignant cells was influenced also by methylation and histone modifications, induced by a histone deacetylase inhibitor (MS-275) and a methylation inhibitor (5-AZC), which proposed that gamma-enolase gene expression is controlled by methylation and histone modifications. The later provides evidence that environmental carcinogenic pollutants, such as cadmium and arsenic, might cause changes in epigenetic regulation of genes, which specifically affect the expression and function of gamma-enolase in breast epithelial cells and urothelial cells.^40^,^49^

## Gamma-enolase in tumour tissues

Gamma-enolase is typically overexpressed in tumours of neurogenic and neuroendocrine origin and has been used as a marker for detection of neuroendocrine differentiation of tumour cells. It is considered the most important tumour marker for poorly differentiated neuroendocrine tumours, since a tumour is classified as a neuroendocrine tumour only when it expresses at least two neuroendocrine markers of which one is gamma-enolase.^80^,^81^ Immunohistochemistry of gamma-enolase is regularly used for differential diagnosis of small-cell lung cancer (SCLC) from other lung cancer histological subtypes (Table 1).^82^,^83^ Gamma-enolase increased expression was observed also in other tumours, including breast cancer, with increased staining in lymph node metastases compared to primary breast tumours^84^ or in glioblastomas, with higher levels in advanced stage tumours, which were related to shorter patient survival.^50^ Nevertheless, immunostaining of gamma-enolase in tumour tissue has limited diagnostic or prognostic utility, since many clinical studies provided contradictory results.^80^,^85^–^87^

## Gamma-enolase in extracellular fluids of cancer patients

In general, gamma-enolase serum levels are better indicators than its tissue expression (Table 1).^80^ Levels of gamma-enolase are elevated in sera from patients with various cancers, however, its appearance in extracellular fluids without any apparent cellular damage is not clear.^1^,^88^ After stroke, brain injury or cardiac arrest, gamma-enolase is released into the cerebrospinal fluid and eventually into the bloodstream due to damage or death of neuronal cells or impairment of the blood-brain barrier integrity. For instance, levels of gamma-enolase in cerebrospinal fluid and serum have been used as a biomarker of cerebral injury and for the assessment of neurological disorders.^38^,^89^,^90^ Gamma-enolase is the only neuroendocrine tumour marker, which is used as a serum marker for follow up and monitoring of therapy effectiveness. Increased gamma-enolase levels in extracellular fluids are related to cancer progression and are typical for cancer in advances stages with distant metastases.^8^,^39^,^80^,^84^–^87^ The levels of gamma-enolase in non-treated cancer increase proportionally to the tumour mass, stage and number of metastases and are related to worse prognosis, however, the levels are not related to the location of metastases.^39^

Gamma-enolase is used in clinical practice in patients with SCLC and neuroblastoma. Its levels are significantly elevated compared to healthy subjects; however, specificity and sensitivity are too low to be used in screening.^39^,^91^ According to the recommendations of expert groups for the use of markers in lung cancer, gamma-enolase is recommended as an auxiliary marker in SCLC for differential diagnosis when biopsy is not possible and when other neuroendocrine tumours are excluded. Further, it is recommended for SCLC postoperative surveillance, for monitoring of therapy in advanced disease and for detection of recurrent disease.^83^,^91^ During chemotherapy, a transient rise of gamma-enolase serum levels occurs due to cytolysis of tumour cells, which disappears in case of successful treatment. However, persistently elevated levels show unsuccessful therapy. Gamma-enolase is not a recommended tumour marker in neuroblastoma; however, it is frequently used for differential diagnosis of neuroblastoma from nefroblastoma and for disease monitoring.^8^,^91^

Gamma-enolase is used as an auxiliary serum marker for follow-up and monitoring of therapy effectiveness in patients with carcinoids, melanoma, seminoma, feocromocitoma, medullary thyroid carcinoma, and endocrine pancreatic tumours. In patients with brain tumours, the levels of gamma-enolase in sera are not elevated, however, increased levels were reported in cerebrospinal fluid.^39^,^91^

Increased serum levels of gamma-enolase were reported also in patients with cancers of nonneuroendocrine origin, such as T-cell leukaemia^92^, B-cell lymphoma^93^ and malignant melanoma.^94^ In general, higher serum levels of gamma-enolase are related to worse prognosis and are the highest in patients with advanced metastatic stage.^39^

Gamma-enolase is usually measured in serum samples and less frequently in cerebrospinal fluid, pleural exudate or ascites. Its half-life in serum is estimated to be approximately 30 h.^101^ The αγ isoform is expressed in large amounts also in erythrocytes and in platelets, therefore it is important to separate blood cells from plasma or serum within 60 minutes from sample collection to prevent haemolysis of blood samples, which could lead to falsely elevated levels of gamma-enolase.^80^,^102^,^103^ Falsely elevated serum levels of gamma-enolase might be also due to various noncancerous pathological causes^104^, such as benign pulmonary diseases^105^, renal failure^106^, brain injuries, seizures, stroke^38^,^107^, severe hypoglycaemia^108^, benign liver diseases^109^ or systemic sclerosis.^110^

## Concluding remarks

Glycolytic enzymes were shown to exert various specific regulatory functions and to play a pleiotropic role in physiological and pathological processes. Therefore, their participation to accelerated glycolysis could not be the only contribution to tumour progression.^2^ Alpha-enolase, the most exhaustively studied enolase isoform, was found to be one of the most frequently altered proteins in human pathologies and suggested as a universal cellular sensor that responds to multiple stimuli and reacts through multiple mechanisms.^6^,^111^ Gamma-enolase, sharing high-sequence identity with alpha-enolase, is also emerging as a multi-functional molecule. Different cellular localisation and interactions with other molecules strongly suggest its multiple cellular engagements.

Gamma-enolase primary role in cancer is the participation to the accelerated glycolysis, which supports increased tumour cell metabolic demands and enables their proliferation. Its C-terminal end might protect tumour cells from stressful conditions and action of therapeutic agents by activating survival-promoting signalling pathways and regulating apoptosis. An additional role of gamma-enolase in cancer progression is its involvement in actin remodelling and consequently in promotion of migration and invasion of tumour cells. These findings suggest that the role of this well-known tumour marker, whose expression is altered during development and progression of a variety of cancers, is pleiotropic and still has to be defined. Future work should be focused on elucidation of gamma-enolase cellular redistribution, interactions with other molecules and involvement in cell signalling. Understanding these processes, together with the tools enabling effective inhibition of gamma-enolase glycolytic activity, might provide new opportunities for cancer treatment.

## Figures and Tables

**TABLE 1.** Use of gamma-enolase as a tumour marker

	**Neuroendocrine cancer**	**Proposed use**	**Use in clinical practice**	**Recommendations**	**Reference**
**Tumour tissues**	SCLC	Differential diagnosis from other lung cancer subtypes	Yes	EGTM, NACB	^[82, 83]^
	Other neuroendocrine tumours (neuroblastoma, endocrine pancreatic tumours, seminoma, medullary thyroid carcinoma, phaeochromocytoma, ect.)	Diagnosis or detection of neuroendocrine differentiation of tumour	Yes		^[80, 81, 95, 96]^
**Serum**	SCLC	Differential diagnosis from other lung cancer subtypes when biopsy is not possible	Yes	EGTM, NACB	^[82, 83]^
		Prognosis	Unknown		^[82, 83, 97]^
		Post-operative surveillance	Yes	EGTM, NACB	^[82, 83]^
		Monitoring efficacy of therapy	Yes	EGTM, NACB	^[82, 83]^
		Detection of recurrent disease after primary surgery	Yes	NACB	^[82, 83]^
	NSCLC	Monitoring therapy in advanced disease	No		^[83]^
		Prognosis	Unknown		^[83]^
	Testicular cancer (seminoma)	Diagnosis	Experimental	EGTM	^[98]^
	Carcinoids	Diagnosis	Unknown		^[96, 99]^
		Monitoring efficacy of therapy	Yes	EGTM	^[8, 39]^
		Detection of early relapse	Yes		^[8, 39, 96]^
	Medullary thyroid carcinomas	Monitoring efficacy of therapy	Yes	EGTM	^[8, 39]^
		Detection of early relapse	Yes		^[8, 39]^
	Phaeochromocytoma	Monitoring efficacy of therapy	Yes	EGTM	^[8, 39]^
		Detection of early relapse	Yes		^[8, 39]^
	Endocrine pancreatic tumours	Diagnosis	Yes		^[95, 96]^
		Monitoring efficacy of therapy	Yes	EGTM	^[8, 39]^
		Detection of early relapse	Unknown		^[8, 39, 99]^
	Paraganglioma	Diagnosis	Unknown		^[99]^
	Neuroblastoma	Differential diagnosis	Unknown		^[8]^
		Prognosis	Yes	ACS	^[100]^
		Monitoring efficacy of therapy	Yes	EGTM	^[8, 100]^
		Detection of recurrent disease	Yes		^[97]^

ACS = American Cancer Society; EGTM = European Group for Tumour Markers; NACB = National Academy of Clinical Biochemistry; NSCLC = non-small-cell lung cancer; SCLC = small-cell lung cancer
